# Doctors’ perspectives on the quality of medical imaging in public hospitals in eThekwini District

**DOI:** 10.4102/hsag.v29i0.2389

**Published:** 2024-05-07

**Authors:** Nkululeko P. Gam, Maureen N. Sibiya

**Affiliations:** 1 Centre for Quality Promotion and Assurance, Faculty of Health Sciences, Durban University of Technology, Durban, South Africa; 2 Division of Research, Innovation and Engagement, Mangosuthu University of Technology, Durban, South Africa; 3 Faculty of Health Sciences, Durban University of Technology, Durban, South Africa

**Keywords:** medical imaging, radiology services, doctors, physicians, clinicians, experiences, challenges, quality assurance

## Abstract

**Background:**

There is a paucity of literature on perspectives of referring doctors about the quality of medical imaging services and this study closes this gap in literature.

**Aim:**

This quality assurance (QA) study aimed to explore the perspectives of doctors on the quality of medical imaging services in selected regional hospitals within eThekwini District of KwaZulu-Natal.

**Setting:**

The study was conducted in four public regional hospitals.

**Methods:**

An exploratory descriptive qualitative research design involving 30 min–45 min of in-depth individual interviews was used. A purposive sampling technique was used to select research participants and hospitals to ensure adequate responses to the research questions. The sample involved nine participants and was guided by data saturation. Responses were recorded through notes and voice recordings and thematic analysis was used to analyse data.

**Results:**

Three main themes (timeliness of examinations, communication and radiology reports and image quality) and eight subthemes (waiting times, shortage of radiographers, workload, communication between doctors and radiographers, requisition forms, unavailability of radiology reports, clarity of images and image acquisition protocols) emerged from the data. Challenges experienced were exacerbated by high workload and shortage of radiologists and radiographers. Doctors in the data collection sites were mainly dissatisfied with services provided by the medical imaging departments.

**Conclusion:**

Regular engagements between medical imaging departments and doctors are important in enhancing the provision of quality care to patients. In-service training of radiographers and employment of additional radiographers and finding solutions to mitigate shortage of radiologists are recommended.

**Contribution:**

This quality assurance (QA) study focused on experiences of doctors while many other medical imaging QA studies in South Africa are equipment based. In-service training of radiographers is recommended to improve image quality and communication skills.

## Introduction

There is a paucity of literature outlining the perspectives of referring doctors on services provided by medical imaging departments. These perspectives are very important for medical imaging departments to improve the quality of their practices. Other methods used by medical imaging departments to address quality matters include performance of routine mandatory quality control (QC) tests, surveys on customer satisfaction and use of multidisciplinary team approach (Graham et al. 2019). In addition, medical imaging plays an important role in medicine today, assisting in disease diagnosis and treatment (Annemari 2017). Gathering data on the experiences of doctors regarding their referral of patients to medical imaging department can assist in identifying gaps when rendering medical imaging services. In turn a medical imaging department that is aware of gaps in its services can improve its quality of services. Ultrasonography, x-rays, mammography, computed tomography (CT scans), magnetic resonance imaging (MRI) and nuclear medicine are all examples of medical imaging technologies (Annemari 2017); however, the current study focused on diagnostic imaging services. Diagnostic imaging services are critical in confirming, assessing and documenting the progression of many diseases and their response to treatment (Haidekker 2013).

A well-structured quality assurance (QA) programme should consider doctors as internal clients of an imaging department and regularly gather their experiences on the quality of the imaging services. In addition, doctors measure the quality of imaging departments through the precision and reliability of imaging reports, timeliness of imaging and effectiveness of communication (Ahmed et al. 2022; Joshi 2009). The quality of final outcomes satisfies both internal and external clients (patients); thus, strengthening the quality of the final outcome improves the quality of a system in general (Hill et al. 2020).

Quality assurance assists in maintaining optimal diagnostic image quality while minimising patient risk and distress (Padmini Kumari, Kumar & Babu 2021). Periodic QC tests, preventive maintenance procedures, administrative methods and training are all parts of QA. It also includes continuous evaluation of the imaging service’s efficacy and the ability to initiate corrective actions (Abdulkadir 2020). The primary goal of a radiology QA programme is to ensure that patients are consistently diagnosed promptly and accurately with minimal risks to ionising radiation. This goal will be adequately met by a QA programme that has three secondary objectives: maintaining diagnostic image quality, minimising radiation exposure to patients and staff and being cost-effective (Abdulkadir 2020). When patients encounter long waiting queues, the disease profile advances (Guttmann et al. 2011) and thus patient may demise due to controllable conditions that could otherwise be avoided (Bernstein et al. 2009); hence, long waiting time is the major cause for dissatisfaction in patients and doctors (Umar, Oche & Umar 2011). Furthermore, some medical conditions are acute in nature and thus require urgent diagnosis followed by urgent interventions like surgery (Biondetti et al. 2019). On the one hand, doctors, physicians and clinicians have a primary responsibility to manage patients (Li 2020), and radiographers, on the other hand, have a fundamental responsibility to assist doctors by taking good quality images at a reasonable short time (Etheredge 2011).

The purpose of this study was to explore perspectives of doctors working in the four regional hospitals within the eThekwini district on the quality of medical imaging services. The objectives of the study were (1) to establish perspectives of the referring doctors on turnaround times for services offered by the imaging departments, (2) to assess perspectives of referring doctors regarding image quality and (3) to explore perspectives of referring doctors regarding communication between radiographers and doctors.

Conducting the study was particularly necessary as there was a paucity of literature on the perspectives of referring doctors on quality of the services provided by the imaging departments. Due to the wide range of services offered by the regional hospitals in eThekwini district, these hospitals also serve as training sites for medical imaging students. It is therefore prudent to improve quality in these hospitals as this will also assist in improving clinical training. Furthermore, regional hospitals in the district are a hub of medical imaging services for the entire province and thus are referral hospitals for many hospitals outside the district and the province. For this reason, quality improvement in regional hospitals particularly in the selected district will be meaningful to many patients received from outside the district as well as those coming from neighbouring provinces.

The primary research question required doctors to share their perspectives on the quality of imaging services as obtained from the imaging departments in their respective hospitals.

## Research methods and design

### Study design

An exploratory descriptive qualitative research study with face-to-face, individual, semi-structured, in-depth interviews with doctors was used to collect data.

### Setting

The study was conducted in four public regional hospitals within the eThekwini district of KwaZulu-Natal in South Africa. The selected regional hospitals offer a range of district and regional hospital services including 24 h accident and emergency, general medicine, general surgery, intensive care, orthopaedics, radiotherapy and oncology (offered by one of the hospitals), dental, and ear, nose and throat (ENT). As provincial hospitals, there are free services offered to pregnant and breastfeeding women and children under the age of 6 years. Furthermore, other patients are charged according to a sliding scale depending on their income. These free and low-priced services, among other factors, contribute to high workload in public hospitals.

### Study population and sampling strategy

A purposive sampling technique was used to select research participants from four regional hospitals. Participants were doctors at the rank of medical officer and above and had to be working in public regional hospitals within the eThekwini district for at least more than a year. Medical students and interns as well as doctors working in the private sector were excluded from the study. These criteria were to ensure that participants had adequate experiences to answer the research questions. Purposive sampling was used to select participants from four of the seven regional hospitals in the eThekwini District to align participants to the aim and research questions (Campbell et al. 2020), with the aim to increase trustworthiness of the results. Sampled doctors were working in Emergency Care units, operating theatres, wards and outpatient departments and they request imaging examinations daily and thus would be able to answer the research questions. In addition, some of the participants specialised in emergency medicine, orthopaedics, surgery and internal medicine.

### Data collection

Data were collected using an interview guide and the primary research question requested doctors to share their perspectives on the quality of medical imaging services. The questions were aligned to the primary research question and research objectives. Pretesting was performed by conducting interviews with two doctors working in a district hospital; thus, responses obtained from it were not included in the study results and the reason for pretesting was to identify unclear statements, thus ensuring that relevant responses would be received from the participants. No corrections were necessary to the data collection tool following pretesting. The data collection tool included a demographic data section and a grand tour question eliciting perspectives of participants on the quality of imaging services offered by the imaging departments. In addition, there were probing questions on specific aspect of service delivery, and these were to ensure that the objectives were adequately achieved. Interviews were performed in English as the researcher and all the participants were proficient in the use of the language. Participants were also requested to suggest strategies that could be used to improve the quality of imaging services. The researcher began each interview with a general introduction including an explanation of the study purpose and indirect benefits of the research to the participants and healthcare services at large. The introduction was beneficial in relaxing participants and explanation of the benefits helped in minimising inaccuracies in the interview data. The interviews were recorded and transcribed verbatim by the researcher following granting of permission by the participants. Probing was used where necessary to obtain in-depth and rich data during interviews. Data collection continued until data saturation and continued further with two more participants beyond data saturation point to ensure that no further new information would emerge (Creswell 2014). Interviews were scheduled for times convenient to both the participants and the imaging departments to avoid disruptions to service delivery. The radiography manager in each hospital was requested to allocate a private room to ensure a conducive environment to conduct interviews. About 30 min – 45 min were allocated for each interview.

### Data analysis

When all recordings of the interviews were transcribed by the primary researcher, the researchers read the transcriptions to become familiar with the data (Creswell 2009). This followed a rigorous process of thematic analysis. Thematic analysis is the process of identifying patterns or themes within qualitative data (Green & Thorogood 2018). All the stages of thematic analysis including developing themes, coding, elaboration and interpretation and checking were followed.

### Trustworthiness of the study

This study was assessed using the criteria for developing the trustworthiness of qualitative research, namely, credibility, dependability, confirmability and transferability. Credibility was ensured by ensuring that the information received from participants was original, and the original views of the participants were correctly interpreted. For this reason, the researchers used quiet spaces and ensured that the participants were relaxed and comfortable in speaking their minds to the researchers. In addition, during the interviews, the researchers would bounce back with participants whatever they have said to ensure similar understanding of statements made. To ensure dependability, the recordings were kept, and field notes were also used to increase reliability. To accomplish confirmability, participants were requested to read the findings of the study to confirm the credibility of the study and its findings. Lastly, to ensure transferability, researchers explicitly and thoroughly described the research setting and the participants.

### Ethical considerations

Ethical approval was obtained from the Institutional Research Ethics Committee of the Durban University of Technology in KwaZulu-Natal prior to data collection (ethical clearance number: IREC 118/19), Prior to ethical clearance permission was sought and obtained from hospital managers of the relevant regional hospitals, eThekwini Health District Manager and KwaZulu-Natal Department of Health. Participants were informed at recruitment stage that their participation would be voluntary and that they would not be harmed in any form (physically, socially, psychologically or otherwise). An in-depth explanation about the study was provided to participants, and informed consents were administered to and signed by those who agreed to take part in the study. The information letter explained that data would be kept in a password-protected computer and would only be accessible to the researchers. Furthermore, confidentiality and the option to withdraw at any stage of data collection were also explained. Hospitals and participants were not mentioned by name but instead were coded for anonymity purposes.

## Results

The results are provided below, and these include demographic details of participants as well as perspectives of doctors on image quality.

### Demographic profiles of the study participants

As illustrated in Table 1, data were collected from nine doctors, and most of the participants were from Hospital A (*n* = 4), and six were females. The plan was to collect data from a minimum of two participants in each hospital, but after more than one visit to one of the hospitals, only one participant was available due to a high patient volume; however, this was acceptable to the researchers as data obtained from most of the hospitals were similar. In Hospital A, there were many participants available; however, two of them provided information beyond data saturation point. Participants held different ranks and had a range of specialisations.

**TABLE 1:** Demographic profiles of the participants.

No	Gender	Race	Hospital	Specialisation	Rank
D1	Male	White	Hospital A	None	Medical officer
D2	Female	White	Hospital A	Emergency medicine	Senior medical officer
D3	Male	Black	Hospital A	Orthopaedics	Registrar
D4	Female	Black	Hospital A	Surgery	General surgeon
D5	Female	Indian	Hospital B	Internal medicine	Medical officer
D6	Female	Black	Hospital C	Internal medicine	Specialist surgeon
D7	Male	Indian	Hospital C	Surgery	Medical officer
D8	Female	Indian	Hospital D	General surgery	Medical officer
D9	Female	Black	Hospital D	General surgery	Medical officer

### Doctors’ perspectives on the quality of medical imaging services

Three main themes and eight subthemes emerged from the data. The main themes were timeliness of examinations, communication as well as radiology reports and quality of images. Subthemes are illustrated in Table 2.

**TABLE 2:** Emerging themes and subthemes.

Themes	Subthemes
1.1 Timeliness of examinations	1.1.1 Waiting times
	1.1.2 Shortage of radiographers
	1.1.3 Workload
1.2 Communication	1.2.1 Communication between doctors and radiographers
	1.2.2 Requisition forms
1.3 Radiology reports and quality of images	1.3.1 Unavailability of radiology reports
	1.3.2 Poor quality images
	1.3.3 Image acquisition protocols

Themes and their respective subthemes are further described below and supporting excerpts are also provided.

## Timeliness of examinations

### Waiting times

Participants expressed concerns regarding the length of time taken by patients in the x-ray department as well as time taken to perform ward and theatre image. These concerns are illustrated by the following excerpts:

‘Most of the time we struggle to get bedside x-rays.’ (D1, Hospital A, Medical Officer)

‘[*F*]or bedside x-rays, because I also work in High Care and we have patients who are bedridden and immobile that need bedside x-rays. Sometimes you want to make a decision about whether you can move a patient from High Care to ICU and that patient might go to ICU without any confirmation because x-rays would only be done after 24 hours. Another example is when you insert a tube and ask for an x-ray to see if the tube is in the correct position and that is usually urgent as you need to take a decision’. The waiting times in this hospital are extremely long and can take up to six hours or more.’ (D7, Hospital C, Medical Officer)

On a positive note, one of the participants was impressed with a quick services rendered by the x-ray department as illustrated below:

‘Our images are sent electronically and these are received very quickly from the x-ray department.’ (D5, Hospital B, Medical Officer)

### Shortage of radiographers

Participants raised the shortage of radiographers as the major contributing factor to poor service delivery. The following excerpts illustrate the concerns raised by the participants:

‘The X-ray department needs to hire more radiographers especially after hours, you need to have at least more than one radiographer working at a particular time.’ (D1, Hospital A, Medical Officer)

‘Most of the time radiographers are not available. We are always told that there is a staff shortage. Radiographers should be available when we need them in theatre so that we don’t ask our interns to screen for us.’ (D3, Hospital A, Orthopaedic Registrar)

‘They must employ more radiographers. I think there are very few of them, sometimes you request for a BSU and you discover there is only one or two people working.’ (D7, Hospital C, Medical Officer)

‘I think there is shortage of radiographers because one person does the whole hospital at times.’ (D6, Hospital C, Specialist Surgeon)

### Workload

The participants also shared their perspectives on the reasons for high workload experienced by staff in the medical imaging departments. Some of the factors stated were ambiguous roles for various levels of healthcare delivery, staff shortages, equipment faults, high patient volumes and unnecessary imaging requests. These expressions are illustrated in the following excerpts:

‘[*B*]ecause the x-ray department services the entire hospital after hours so there can be quite substantial delays.’ (D2, Hospital A, Senior Medical Officer)

‘The problem is there are high patient volumes and many patients that are not supposed to go for x-rays do go for x-rays especially after hours. …. That unfortunately leads to an increased overload and strain on one or two radiographers that are available after hours.’ (D8, Hospital D, Medical Officer)

‘And then there must be compassion for fellow healthcare workers. We must understand that after hours and weekends there is skeletal staff so we should not request x-rays to assess chronic conditions because the few people that are there get overworked. The reason for all of this is because the general population cannot understand what emergency is and what is not emergency.’ (D9, Hospital D, Medical Officer)

## Communication

### Communication between doctors and radiographers

One of the participants expressed that they did not always receive the images requested and radiographers lacked communication skills. The concern is illustrated by the excerpt below:

‘Radiographers need to communicate more with us. On the x-ray request form you write the history that the patient presented with together with examination findings. Then you request erect abdominal x-ray because you know what you want, but the patient comes back with chest x-ray, it is not what you requested, you then send the patient back and you are re-exposing the patient.’ (D7, Hospital C, Medical Officer)

‘You request a particular view in line with patient history and that radiographer does something else without discussing the request with you.’ (D3, Hospital A, Orthopaedic Registrar)

### Requisition forms

The participants expressed their dissatisfaction with requisition forms used when requesting x-ray examinations. One of the participants was satisfied with paper-based and online request forms used in their hospital while they also expressed some disadvantages regarding the paper-based request form. The following excerpt illustrates their expression:

‘The x-ray department has an online form that we complete but in cases of load shedding and other instances we use a paper-based request form. The advantage of the online form is that it provides a drop-down menu with all the different views you can select from and it is much quicker than completing the hard copy one.’ (D1, Hospital A, Medical Officer)

An interesting finding was that in a hospital where the radiology services are predominantly digital, a paper-based request form was used. A participant in this hospital also expressed dissatisfaction about the layout of the request form and his concerns are illustrated by the following excerpt.

‘The x-ray department staff should improve the form itself. I mean the form is very old and they must have been using it for almost 30 years or more. A digital system of ordering x-rays will help a lot. I used to work in private and online requisitions are used instead of a manual one. They should also consider changing the format of the form.’ (D5, Hospital B, Medical Officer)

## Radiology reports and quality of images

### Unavailability of radiology reports

Many participants were concerned about the unavailability of radiology reports. The following excerpts serve as illustrations:

‘We don’t get reports at all, but also to be fair, I don’t think it would be feasibly for a radiologist to report on all the x-rays we request as there are too many. However, reports are available on request. The reports are very much consultant dependent and some of us would go down to discuss them with the radiologist.’ (D2, Hospital A, Senior Medical Officer)

‘We do not receive the reports due to shortage of staff. Unfortunately, we have to interpret x-rays ourselves. Reports may be requested but we hardly request them because it takes time to get the report.’ (D4, Hospital A, General Surgeon)

‘We do not receive x-ray reports. Unfortunately, this affects service delivery. The time it takes me to read an x-ray of which I’m not a specialist at, can be used to assess another patient. The other side of the story is that, because I am not a specialist in reading x-rays, there are things that I might miss that a specialist radiologist would pick up. So, there is also a possibility of misdiagnosis.’ (D8, Hospital D, Medical Officer)

‘In our department (orthopaedic), we do not usually request reports probably due to our level of experience. We simple interpret them ourselves you know. We occasionally request for a report, especially in the area of spine examinations. We do need reports for malignancies though.’ (D3, Hospital A, Orthopaedic Registrar)

‘As doctors in the Accident and Emergency department we are used to interpret the x-rays on our own. We do not really need the reports to manage the patients because we just make the assessment on our own.’ (D5, Hospital B, Medical Officer)

### Quality of images

Many participants in the current study were dissatisfied with the quality of the images. The participants described poor image quality as poor positioning, lack of or incorrect image identification and imaging of unaffected (unrequested) parts. These are illustrated in the following excerpts:

‘Secondly I think senior radiographers should be paired with junior ones to ensure clarity of images so that patients to not have to visit the x-ray department again for repeats.’ (D3, Hospital A, Orthopaedic Registrar)

‘The clarity of images depends on the staff who took them. There are people who are not well trained. Yesterday I sent a patient to exclude chest infection, but the x-rays were so poor that I could not use them in decision making. I had to take bloods to exclude the infection because the x-ray was very faint.’ (D7, Hospital C, Medical Officer)

‘Junior staff must be trained to take proper x-rays. Sometimes you send patients to x-ray during working hours and then the patient comes back with poor quality x-rays or sometimes a wrong examination.’ (D7, Hospital C, Medical Officer)

‘Radiographers must do the images that we request because you ask for a radius and then they just do wherever the patient says is painful. They also need to print good quality films, not over penetrated or under penetrated.’ (D7, Hospital C, Medical Officer)

‘There are issues with orthopaedic x-rays that we order. If we request specific x-rays we don’t get those x-rays. It is simple things like if you request a specific joint like an ankle, the x-ray must be centred at the ankle, but we end up with an x-ray of the entire lower limb.’ (D2, Hospital A, Senior Medical Officer)

‘Patients must be correctly identified so that I do not read an x-ray that actually belongs to another patient. Because it does happen that you send Mr Mnguni to x-rays and you get x-rays of Mr Harripersad.’ (D1, Hospital A, Medical Officer)

On the positive side, some participants were satisfied with the quality of images as expressed in the following excerpts:

‘The x-rays we get are quite good compared to the quality we get from the private sector where I work the other four days in a week. I occasionally request for repeats to be done but overall the images are quite good.’ (D4, Hospital A, general Surgeon)

‘The images produced by the x-ray department are very good.’ (D5, Hospital B, Medical Officer)

### Image acquisition protocols

The participants complained that there was no image acquisition protocol communicated to them and they were not sure of what to do to request some specialised examinations like the CT scan. Too many images in computed radiography (CR) were printed on one film when the correct sizes were not available. The following excerpts illustrate the responses:

‘I have never seen a protocol for acquisition of mages.’ (D4, Hospital A, General Surgeon)

‘We don’t have a protocol regarding x-rays. We go by clinical judgement, then we call for special investigations like CT scan to discuss with the radiologist then we book the examination. You are not allowed to simple fill a form and send a patient, no you’ve got to discuss each case with the radiologist.’ (D8, Hospital D, Medical Officer)

‘Sometimes more than one images get squeezed in one film and they are very small. You find there is a pelvis, there is a knee, and there is an ankle in one film. It makes it difficult to diagnose properly.’ (D3, Hospital A, Orthopaedic Registrar)

## Discussion

The purpose of the study was to explore perspectives of referring doctors on diagnostic imaging services. Themes emerging from the data included timeliness of images, communication and radiology reports and quality of images. The findings showed that interviewed doctors working in the data collection sites were dissatisfied with many aspects of the diagnostic imaging services. The challenges included inadequacy of request forms, poor communication between radiographers and referring doctors, long waiting times and poor image quality. In addition, the challenges experienced were exacerbated by the high workload and a shortage of radiologists and radiographers.

The results of the current study are similar to a previous study conducted in primary healthcare facilities in Saudi Arabia (Alsharafi & Mokali 2020) in which referring doctors were dissatisfied with many aspects of the diagnostic imaging services including the shortage of radiologists and radiographers, unavailability of radiology reports and unavailability of diagnostic imaging equipment. On the other hand, the results of the current study contradict a previous study (Jossen et al. 2019) in which the referring doctors were satisfied with diagnostic imaging services. The results from the latter study (Jossen et al. 2019) provide hope that the circumstances currently prevailing in regional hospitals in the eThekwini hospitals can be changed such that doctors become satisfied with imaging services.

Many explanations can be made about the results of the current study. Communication between radiographers and referring doctors is facilitated using a request form. The form should be designed such that it provides the imaging department with all the necessary information that will help the radiographer and the radiologist to focus on the area of interest. The doctor-radiographer interaction is thus important to enhance the patient experience through the multi-level referral systems (Makanjee, Bergh & Hoffmann 2015). It is, therefore, important for radiographers to seek clarity from the referring doctors when the request forms are lacking the necessary details. The guidelines by the South African Health Products and Regulatory Authority (SAHPRA) on requests for medical imaging examinations (SAHPRA 2022) prescribe effective doctor-radiographer communication to ensure that radiation dose to the patient is optimal.

Radiographic images should possess characteristics suitable for them to assist in patient diagnosis (Cândido et al. 2013) as poor quality images are of no benefit to the patients and thus patients are simply exposed to unnecessary radiation. Furthermore, there is a linear relationship between image quality and satisfaction of the referring doctor (Utami 2020), and thus ‘unclear’ images as reported in the current study should be avoided. Probing of participants revealed that ‘unclear’ images lacked visibility and detail due to poor image contrast and density. These can be improved by setting of correct exposure factors and use of other imaging modalities such as CT and ultrasonography (Lampignano & Kendrick 2020). In addition, in-service training on patient positioning and manipulation of exposure factors should be conducted among radiographers to improve their skills (Sebelego, Van Der merwe & Du Plessis 2019) to clearly show anatomical structures. A surprising finding was that participants had to request other diagnostic tests like blood tests due to poor image quality. The ramifications of this finding are the reduced patient satisfaction and unnecessary extra costs for the state (Gijo & Antony 2014).

In addition, the unavailability of image acquisition protocols (or lack of its communication to the referring doctors) needs to be urgently addressed as this is a requirement of the quality standards for imaging (Hertel & Chang 2007). The image acquisition protocol would guide junior radiographers and doctors of the projections to be taken to adequately demonstrate anatomical structures and relevant techniques of each requested anatomical part. In addition, it can also be customised to include the number of images to be printed in each image receptor. These protocols should be approved by the radiologists and must be communicated to all the radiographers and referring doctors within the hospitals.

Furthermore, the finding that doctors had to wait for many hours for patients referred to the imaging department to return is a major concern for both patients and doctors as it has implications on the management of patient conditions. It is surprising that there were negative comments on turnaround times as a significant improvement was expected since digital radiography was used in most of the imaging facilities (Nitrosi et al. 2007). Hospital management should take reasonable steps to improve patient-centred care and all stakeholders must be well trained on such initiatives (Hyde & Hardy 2021). One of the strategies to improve turnaround times as suggested by the participants in the current study is to employ more radiographers and radiologists. According to the Health Professions Council of South Africa (HPCSA), there are currently close to 8000 radiographers and nearly 1000 radiologists in South Africa, and these numbers (especially radiologists) are too low to cater for more than 60 million people in South Africa. In addition, delay or unavailability of image reports can result in medical errors which can in turn result in avoidable patient deaths (Donnelly et al. 2010) as some of the healthcare providers are unable to interpret radiographic images on their own (Semakula-Katende, Andronikou & Lucas 2016). One of the options to make reports available would be to train radiographers to report on images but many radiographers are sceptical about producing a written image report (Kekana, Swindon & Mathobisa 2015). It must also be remembered that a change in the scope of practice for radiographers in South Africa is needed before reporting can be implemented. On the other hand, several studies support the notion of radiographer reporting (Wuni, Courtier & Kelly 2020; Gqweta 2012; Buskov et al. 2013). A comprehensive stakeholder agreement needs to be reached to foster a successful radiographer reporting initiative in South Africa. Such an agreement would involve identification of training gaps, development of a relevant curriculum and a revision of enrolment figures for radiography students by universities to ensure that the imaging services are not negatively affected while trying to address image reporting challenges.

The strength of the study was participants with varying experiences from multiple hospitals, thus providing a holistic view of the experiences. This was to ensure that while generalisation of findings is limiting in qualitative research, the findings from the current study are at least applicable to the regional hospitals in the eThekwini District. In addition, the use of one-on-one, in-depth interviews provided the researchers an opportunity to ask probing follow-up questions, thus obtaining interpretation of results from the participants themselves and not the researchers.

Limitations in research are defined as restrictions that the researcher has no control over (Theofanidis & Fountouki 2018). A limitation to the current study is that it was undertaken in public hospitals and participants from the private sector could possibly provide differing views. Focus group discussions with medical consultants from various specialties within the hospitals could be used to further enhance the richness of the data. Radiographers and radiography managers were also interviewed to assess factors affecting quality within medical imaging departments, and the results of that study will be presented in a different report. In addition, from the results of the current study, there is a need to conduct a follow-up study to investigate the reasons for the possible inferior image quality and also provide in-service training to radiographers. It is however appreciated that the study added an important value, and its results can be used by the decision-makers to improve medical imaging services in regional hospitals within the eThekwini District.

## Conclusion

The researchers were able to achieve the aim and objectives of the current study as perspectives of referring doctors were sufficiently explored and suggestions to address them were also made. The take-home message from this study is that as main stakeholders in imaging services, referring doctors must be included in endeavours to improve the quality of imaging departments. Perspectives of referring doctors are highly important as they are the recipients of the services provided by imaging departments. While patient satisfaction relates to how they are treated in the imaging department, satisfaction of doctors on the other hand is concerned with image quality, communication and timelines of services, and these play a different role to QA compared to patient satisfaction.

The need for in-service training of radiographers and employment of more radiographers are the main solutions to the challenges experienced by referring doctors in the eThekwini District of KwaZulu-Natal. Anecdotal evidence depicts a bad picture regarding shortage of radiographers in public regional hospitals within the eThekwini District. This is supported by a comment made by the participants in the current study who unanimously agreed that there is a severe shortage of radiographers impacting on service delivery.

Future research should consider replicating the study and involve participants from both public and private sectors. The researchers are of the view that a similar study involving doctors working in private practices could provide different information due to the differences in working conditions and availability of resources.
